# Studying the Optical 3D Accuracy of Intraoral Scans: An In Vitro Study

**DOI:** 10.1155/2020/5739312

**Published:** 2020-02-14

**Authors:** Pokpong Amornvit, Sasiwimol Sanohkan, Chaimongkon Peampring

**Affiliations:** Department of Prosthetic Dentistry, Faculty of Dentistry, Prince of Songkla University, Hat Yai, Songkhla 90110, Thailand

## Abstract

There are various scanners available in dental practice with various accuracies. The aim of this study was to compare the 3D capturing accuracy of scans obtained from Trios 3 and Dental Wings scanner. A reference mandibular model was printed from FormLab with reference points in three axes (*X*, *Y*, and *XY* and *Z*). The printed model was scanned 5 times with 3 scans: normal scan by Trios 3 (Trios 3A), high-resolution scan by Trios 3 (Trios 3B), and normal scan by Dental Wings. After scan, the stereolithography (stl) files were generated. Then, the measurements were made from the computer software using Rhinoceros 3D (Rhino, Robert McNeel & Associates for Windows, Washington DC, USA). The measurements made with digital caliper were taken as control. Statistical analysis was done using one-way ANOVA with post hoc using Sheffe (*P* < 0.01). Trios 3 presented higher accuracy than Dental Wings and high resolution showed better results. The Dental Wings showed less accuracy at the measurements >50 mm of length and >30 mm in width. There was no significant difference (*P* > 0.05) of control with the Trios 3A and Trios 3B. Similarly, for the measurements in *Z*-axis, there was no significant difference of control with each scan (Trios 3A, Trios 3B, and Dental Wings). Accuracy of the scan is affected by the length of the scanning area and scanning pattern. It is less recommended to Dental Wings scan >3-unit prosthesis and that crosses the midline.

## 1. Introduction

There has been massive advancement in digital dentistry in the recent decade, especially since the invention of computer-aided design/ computer-aided manufacturing (CAD/CAM) system, milling systems, rapid and automated prototyping, and three-dimensional (3D) printing of dental biomaterials, and these have revolutionized and created a new modality in dentistry [1]. Currently, CAD/CAM is widely used in virtual occlusal records, full-mouth reconstruction, and orthodontics [2–4]. Moreover, they are extensively used in both the dental laboratory and the dental clinic for the fabrication of various prosthesis, such as inlays, onlays, veneers, crowns, fixed partial dentures, orthodontic aligners, surgical guides, and implant abutments [3]. A 3D scanning is a process that is used to capture the shape of an object using a 3D scanner. After scanning, a 3D file of an object is created which can be edited, and 3D printed. An intraoral scan (IOS) can be based on many different technologies, each with its own limitations, advantages, and costs [5]. Many limitations in the kind of objects that can be digitized are still present. For example, optical technology may encounter many difficulties with shiny, reflective, or transparent objects.

Teeth, especially the anterior, play in the esthetics of face [6]. For a successful esthetic dental restoration, a good dental impression is important [7, 8]. With the use of digital dentistry, the intraoral conditions can be transferred digitally and printed. When dental laboratories receive a digital impression, they create a model from the data and either continue with the traditional fabrication procedure or rescan the model and fabricate the prosthesis. The dental technician can do all the design restorative works directly on the computer based on the digital file received. Hence, the digital impression plays an important in the fabrication of all digital works and the accuracy of the impression is very important.

There are various 3D scanners technologies, such as image capturing or video capturing type contact scanning or noncontact scanning [9–12]. Contact canners probe the subject through physical touch while the object is in contact with or resting on a precision flat surface plate, ground and polished to a specific maximum of surface roughness. Noncontact scanners emit some kind of radiation or light and detect its reflection or radiation passing through the object in order to probe an object or environment [11]. Nowadays, the noncontact scanning technique is recommended widely. Types of scanning technology: the 3D scanning technologies rely on different physical principles and are explained in following categories [5]:

1. Laser triangulation 3D scanning technology uses either a laser line or a single laser point to scan across an object.
2. Structured light 3D scanning technology uses trigonometric triangulation but not the laser.
3. Photogrammetry 3D scan scanning technology (photography) reconstructs 3D from 2D captures with computer vision and computational geometry algorithms.
4. Contact-based 3D scanning technology is based on contact form of 3D data collection and uses a contact probe.

Accuracy comprises precision and trueness (ISO 5725-1) [13]. Precision describes how close repeated measurements are to each other [14]. The higher the precision, the more predictable the measurement is. Trueness describes how far the measurement deviates from the actual dimensions of the measured object. A high trueness delivers a result that is close or equal to the actual dimensions of the measured object. Many factors influence the accuracy of the IOS such as [15–17]:

1. Scanner: ability to record details and its accuracy
2. Operator: scanning principles and span of scanning
3. Scanning area: size of scanning area, arch length, and surface irregularities
4. Intraoral environmental factors: temperature, relative humidity, and illumination

The IOS accuracy is enhanced by reducing the span of scanning, and ensuring the scanned surfaces exhibit minimal irregularities [10]. The problem with IOS is that it can be difficult to detect deep margin lines in prepared teeth and/or in case of deep margins or bleeding [18]. In addition, various studies done in evaluating the digital impression highlights several issues such as distortion of the digital models, problems with the intraoral conditions, and lower precision compared to conventional impressions [19, 20]. In addition, digital scanners with high accuracy are currently limited to small measurement fields such as single teeth or quadrants [19, 21]. The aim of this study was to compare the 3D capturing accuracy of scans obtained from Trios 3 and Dental Wings scanner in an in vitro study design.

## 2. Materials and Methods

**Figure 1:**
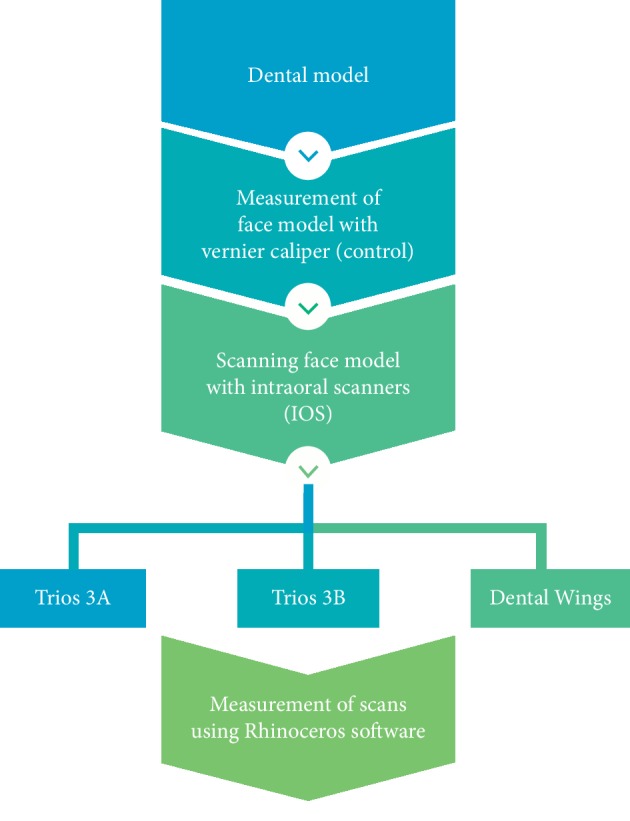
Details of the study overview.

A method modified from the American National Standard/ American Dental Association (ANS)/ADA) Standard No. 132 for the scanning accuracy was used in this study [22]. The study consists of fabrication of dental model, scanning, and measurements. The details of the study are shown in (Figure 1).

### 2.1. Fabrication of Dental Model

**Figure 2:**
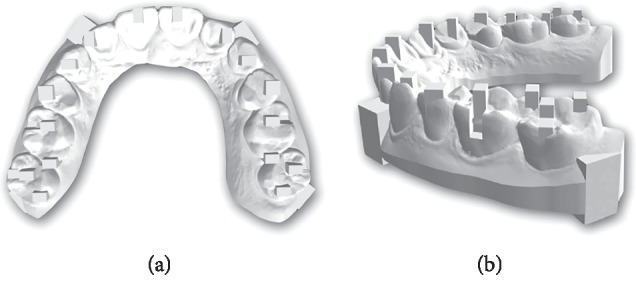
Digital dental model with various points marked in the three axes (*X*, *Y*, and *Z*).

A digital mandibular model is made in the computer. Various points were marked on the digital model where the measurements can be measured in three axes (*X*, *Y*, and *Z*) (Figure 2).

**Figure 3:**
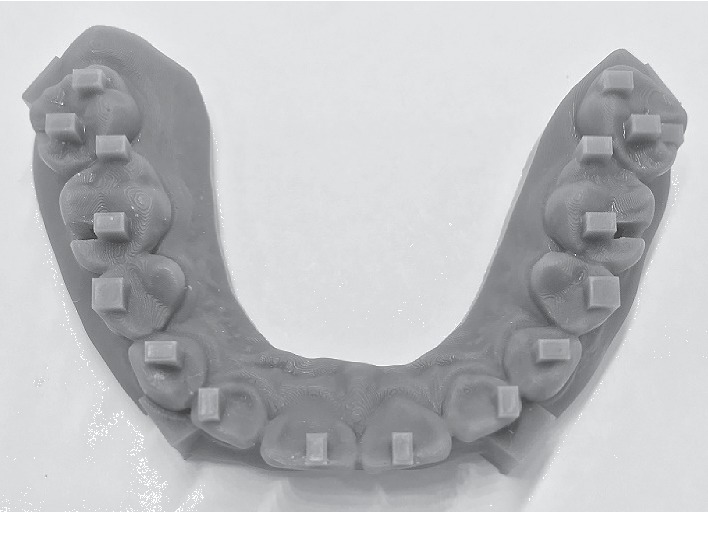
Printed mandibular dental model.

The model was printed using FormLab following manufacturing recommendations. From 3 dental models, the best model was selected for this study as shown in Figure 3.

### 2.2. Scanning

**Figure 4:**
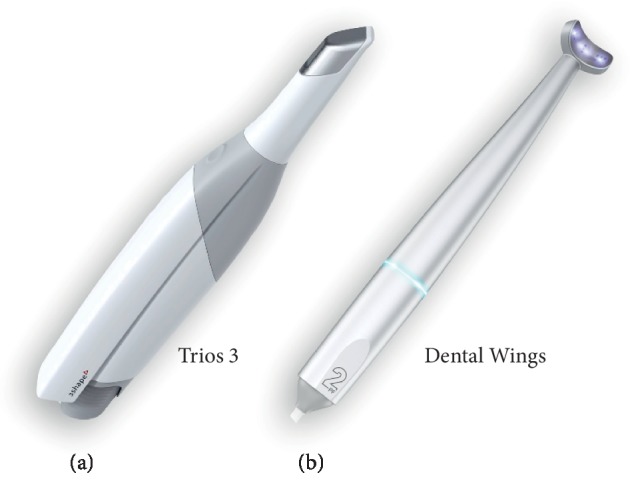
Intraoral scanners (IOS): (a) Trios 3 and (b) Dental Wings.

The printed model was scanned 5 times each with 3 Shape Trios 3A: normal scan, 3 Shape Trios 3B: high resolution (3 Shape Trios A/S 2018, Copenhagen, Denmark), and Dental Wings (Dental Wings Inc., Montreal QC, Canada) (Figure 4) according to the manufacturer's recommendation. After the scan, the scanned files were saved as stereolithography (STL) files.

### 2.3. Measurements

**Figure 5:**
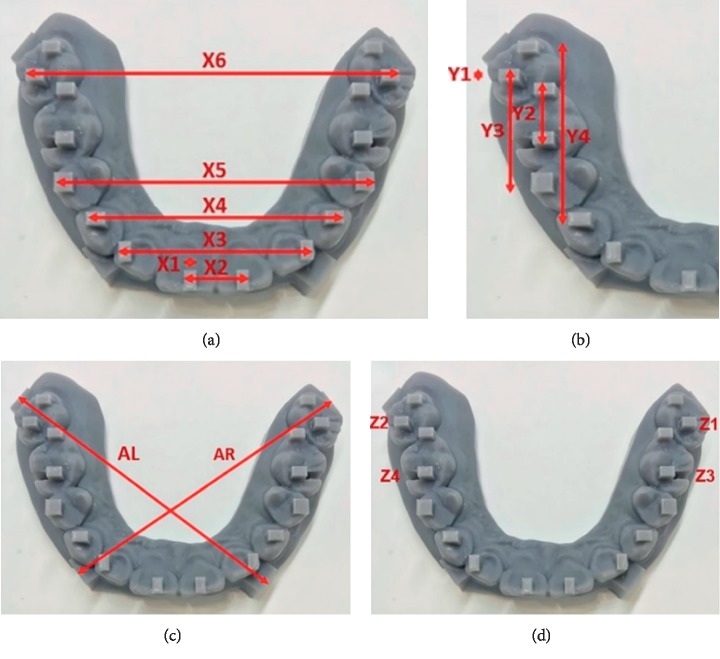
Reference points on the model and various measurements measured on model in different axes: *X*-axis (a), *Y*-axis (b), *XY*-axis (c), and *Z*-axis (d).

**Figure 6:**
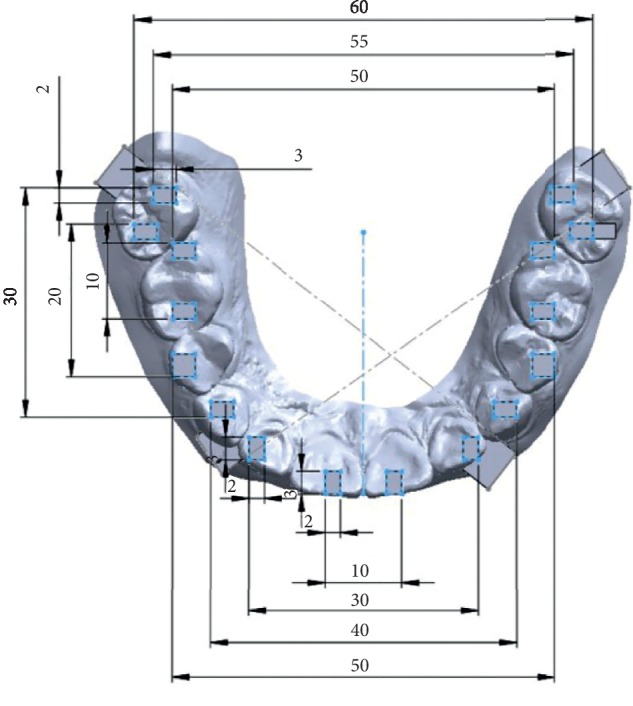
Reference points on the model and various measurements in the *X*-axis (length) and *Y*-axis (length) from software of 1 scan.

Then, for the scanned files, the measurements were made from computer software using the Rhinoceros 3D modeling software (Rhino, Robert McNeel & Associates for Windows, Washington, DC, USA). The measurements were done in 3 axes (*X*, *Y*, and *XY* and *Z*) of various lengths as follows (Table 1 and Figures 5 and 6). The measurements made on the printed model with digital caliper were taken as the control (Figure 5).

In addition, the quality of the scans and capturing details of the both scanners were also evaluated.

### 2.4. Statistical Analysis

Microsoft Excel 2010 and SPSS version 20 (IBM Company, Chicago, USA) were used for the descriptive statistics and expressed as mean and standard deviation. Multiple comparison was done using one-way ANOVA with post hoc using Sheffe to see the significant difference (*P* < 0.01) between the control (dental model) and scans.

## 3. Results

Tables 2–4 shows the descriptive statistics of the scans of various lengths in the three axes (*X*, *Y*, *XY*, and *Z*).

The multiple comparisons between the measurements of dental model and the scan are shown in Tables 5–7. It was seen that there was significant difference (*P* value <0.01) of the measurements *X*1, *X*2, *Y*1, *Y*2, *Y*3, AR, AL, *Z*1, *Z*3, and *Z*4 between the dental model and the scans.

For the measurements in the *X*-axis, *X*1–*X*4, there was no significant difference of each scan (Trios 3A, Trios 3B, and Dental Wings) compared to the control as shown in Table 5. But, *X*5–*X*6, Dental Wings showed there was significant difference (*P* value <0.01) from the dental model (control). Hence, Dental Wings showed less accuracy at the measurement length 50 mm and 60 mm.

Similarly, for the measurements in the *Y*-axis, *Y*2–*Y*4, there was no significant difference of each scan (Trios 3A, Trios 3B, and Dental Wings) compared to the control as shown in Table 6. But, for *Y*1, Dental Wings showed there was significant difference (*P* value = 0.015) from the dental model (control). Furthermore, for the measurements in the *XY*-axis, AR and AL, there was significant difference (*P* value <0.005) of Dental Wings compared to the control. But, there was no significant difference (*P* value >0.05) of control with the Trios 3A and Trios 3B for the AR and AL. Hence, Dental Wings showed less accuracy in the measurements.

Similarly, for the measurements in the *Z*-axis, *Z*1–*Z*4, there was no significant difference (*P*=0.05) of control with each scan (Trios 3A, Trios 3B, and Dental Wings) as shown in Table 7.

Regarding the quality and capturing details, Trios 3A showed the best results followed by Trios 3B and Dental Wings.

## 4. Discussion

Digital impressions reduce the patient discomfort; intraoral scanners (IOS) are time-efficient and simplify clinical procedures for the dentist and the laboratory technician, eliminating plaster models and allowing better communication with the dental technician and with patients. The accuracy of which influences the fit of the restorations, an important factor in the longevity of the final restoration [7, 8]. Renne et al. [23] compared 7 different IOS and they found that the Planscan had the best accuracy (trueness and precision) while the 3Shape Trios was found to be the poorest for sextant scanning. The order of trueness for complete arch scanning was as follows: 3Shape D800 > iTero > 3Shape TRIOS 3 > Carestream 3500 >Planscan > CEREC Omnicam > CEREC Bluecam. The order of precision for complete-arch scanning was as follows: CS3500 > iTero > 3Shape D800 > 3Shape TRIOS 3 >CEREC Omnicam > Planscan > CEREC Bluecam. For the secondary outcome evaluating the effect time has on trueness and precision, the complete-arch scan time was highly correlated with both trueness and precision. They concluded that for complete-arch scanning, the 3 Shape Trios was found to have the best balance of speed and accuracy. Park et al. [15] designed an intraoral environment simulator to assess the accuracy of 2 IOS using the simulator and found no difference due to the intraoral environment. The simulator contributes to the higher accuracy of IOS.

Mutwalli et al. [24] studied the trueness and precision of different IOS when scanning fully edentulous arch with multiple implants. They found that there were significant differences between all IOS. For the implant measurements, Trios 3 had the lowest trueness, followed by Trios 3 mono and Itero element. Trios had the lowest precision, followed by Itero element and Trios 3 mono. Regarding the interarch distance measurements, Trios 3 had the lowest trueness, followed by Trios 3 mono and Itero element. Trios 3 had the lowest precision, followed by Itero element and Trios 3 mono. But, in our study, Trios 3 presented higher accuracy than Dental Wings and high resolution showed better results. There can be minor errors in the measurements by the IOS in various steps. While scanning 1 arch, generally, the IOS captures around 1200 images. Errors in scanning may be due to overlapping of the partial images, especially in the anterior region [19, 25]. The occurrence of more errors of digital impression in the anterior regions is due to the less structured tooth surface and steep inclines. The superimposition process leads to the deviation. These errors might be reduced or avoided with further software improvements. In addition, there can be errors in computer processing, which may be due to filter algorithms and calibration errors of the scanner [25]. There can be errors in the x-axis, y-axis, and z-axis, but in our study, there were more errors in the *z*-axis (depth of scanning). The errors can be avoided by a longitudinal measurement of a calibrated length specimen.

The dimension measured can be implemented as follows. In the anterior region, *X*1 (2 mm) represents a scan body or an onlay, *X*2 (10 mm) represents 1-unit restoration or prosthesis, *X*3 (30 mm) represents the 4-unit restoration, *X*4 (40 mm) represents the 6-unit restoration, *X*5 (50 mm) represents the 10-unit restoration, and *X*6 (60 mm) represents the 14-unit restoration or full-arch restoration. *X*1–*X*4 represent the dimensions in one quadrant in the same arch (upper or lower). *X*5 and *X*6 extend to 2 quadrants in the same arch. Trios 3 allows us to record full-arch and high-resolution scans that are more accurate than normal scans, but there was no significant difference. In addition, Dental Wings allow for maximum 6-unit restorations.

In the posterior region, *Y*1 (2 mm) represents a scan body or an onlay, *Y*2 (10 mm) represents 1-unit restoration or prosthesis, *Y*3 (20 mm) represents the 2-unit restoration, and *Y*4 (30 mm) represents the 4-unit restoration. Similarly, AR and AL (65 mm) represent the full-arch restorations. For posterior measurements, all scanners showed acceptable accuracy. But more than 3-unit restoration showed less accuracy (*P* value <0.05). If we want to fabricate prosthesis with model less, >3-unit restoration is not recommended. For Dental Wings, the accuracy is less for the restoration that crosses the midline; hence, there is a need to be careful. It is less recommended to scan >3-unit prosthesis.

## 5. Conclusion

Within the limitations of this study, Trios 3 presented higher accuracy, better quality, and captured more details than Dental Wings, and high resolution showed better results. Accuracy of the scanners is affected by the length of the scanning area and scanning pattern. It is less recommended to use Trios 3 for scanning >3-unit prosthesis (50 mm) and that crosses the midline.

## Figures and Tables

**Table 1:** Measurements in three axes (*X*, *Y*, *XY*, and *Z*) of various lengths.

*X*-axis	*Y*-axis	*XY*-axis	*Z*-axis
*X*1: Mesiodistal width on teeth #21 (2 mm)	*Y*1: 2 mm buccolingual width on teeth #27 (2 mm)	AR: Diagonal distance from #12 to #27 (65 mm)	*Z*1: Buccal notch on #17 (2 mm)
*X*2: Distance from #11 to #21 (10 mm)	*Y*2: Buccolingual width on teeth #26 (10 mm)		*Z*2: Buccal notch on #27 (4 mm)
*X*3: Distance from #12 to #22 (30 mm)			*Z*3: Buccal notch on #16 (6 mm)
*X*4: Distance from #13 to #31 (40 mm)	*Y*3: Buccolingual width from #25 to #27 (20 mm)	AR: Diagonal distance from #22 to #17 (65 mm)	*Z*4: Buccal notch on #27 (8 mm)
*X*5: Distance from #14 to #41 (50 mm)			
*X*6: Distance from #16 to #61 (60 mm)	*Y*4: Buccolingual width from #23 to #27 (30 mm)		


**Table 2:** Descriptive statistics of measurements of various groups in the *X*-axis.

Measurements	Groups	Mean	SD	95% CI for mean		Min	Max
				Lower bound	Upper bound		
*X*1	Control	1.938	0.039	1.888	1.987	1.90	2.00
	Trios 3A	1.876	0.032	1.836	1.916	1.85	1.93
	Trios 3B	1.886	0.036	1.841	1.931	1.84	1.94
	Dental Wings	1.876	0.057	1.805	1.946	1.83	1.96
	Control	9.938	0.022	9.911	9.964	9.90	9.95

*X*2	Trios 3A	9.864	0.027	9.835	9.892	9.84	9.90
	Trios 3B	9.968	0.028	9.933	10.002	9.92	9.99
	Dental Wings	9.866	0.071	9.778	9.954	9.79	9.97
	Control	29.852	0.06	29.77	29.926	29.78	29.93

*X*3	Trios 3A	29.734	0.022	29.706	29.761	29.70	29.76
	Trios 3B	29.914	0.033	29.872	29.955	29.87	29.95
	Dental Wings	29.806	0.172	29.592	30.019	29.60	30.03
	Control	39.910	0.054	39.842	39.978	39.82	39.96

*X*4	Trios 3A	39.716	0.011	39.702	39.73	39.70	39.73
	Trios 3B	39.698	0.248	39.389	40.007	39.39	39.93
	Dental Wings	40.112	0.177	39.892	40.332	39.88	40.28
	Control	50.03	0.035	49.986	50.074	49.98	50.07

*X*5	Trios 3A	49.86	0.118	49.713	50.006	49.75	50.02
	Trios 3B	50.21	0.136	50.04	50.379	49.98	50.31
	Dental Wings	50.832	0.423	50.306	51.357	50.43	51.55
	Caliper	60.616	0.052	60.552	60.68	60.54	60.67
	Control	60.068	0.271	59.732	60.404	59.66	60.42

*X*6	Trios 3A	60.368	0.514	59.728	61.007	59.77	60.83
	Dental Wings	61.952	0.374	61.487	62.417	61.55	62.41

SD = standard deviation; CI = confidence interval for mean; min = minimum; max = maximum.

**Table 3:** Descriptive statistics of measurements of various groups in *Y*-axis and *XY*-axis.

Measurements	Groups	Mean	SD	95% CI for mean		Min	Max
				Lower bound	Upper bound		
*Y*1	Control	1.932	0.008	1.921	1.942	1.92	1.94
	Trios 3A	1.856	0.035	1.812	1.899	1.80	1.89
	Trios 3B	1.854	0.077	1.758	1.949	1.78	1.97
	Dental Wings	1.828	0.017	1.805	1.85	1.80	1.85

*Y*2	Control	9.876	0.021	9.85	9.901	9.86	9.91
	Trios 3A	9.806	0.149	9.619	9.992	9.54	9.89
	Trios 3B	9.81	0.082	9.708	9.912	9.74	9.93
	Dental Wings	9.8	0.074	9.707	9.892	9.71	9.90

*Y*3	Control	19.934	0.047	19.875	19.992	19.89	20.01
	Trios 3A	19.71	0.12	19.561	19.859	19.56	19.84
	Trios 3B	19.81	0.062	19.733	19.886	19.75	19.90
	Dental Wings	19.636	0.518	18.993	20.278	18.81	20.02

*Y*4	Control	30.162	0.339	29.748	30.575	29.86	30.70
	Trios 3A	29.974	0.391	29.488	30.459	29.38	30.35
	Trios 3B	30.186	0.593	29.45	30.921	29.60	31.05
	Dental Wings	29.754	0.425	29.225	30.282	29.17	30.37

AR	Control	65.126	0.037	65.079	65.173	65.10	65.19
	Trios 3A	64.872	0.119	64.724	65.019	64.78	65.07
	Trios 3B	65.124	0.173	64.908	65.339	64.90	65.32
	Dental Wings	66.044	0.624	65.268	66.819	65.17	66.88

AL	Control	65.094	0.053	65.027	65.16	65.03	65.15
	Trios 3A	65.43	0.484	64.828	66.031	64.88	65.81
	Trios 3B	65.408	0.326	65.002	65.813	64.97	65.89
	Dental Wings	65.942	0.133	65.776	66.107	65.84	66.14

SD = standard deviation; CI = confidence interval for mean; min = minimum; max = maximum.

**Table 4:** Descriptive statistics of measurements of various groups in the *Z*-axis.

Measurements	Groups	Mean	SD	95% CI for mean		Min	Max
				Lower bound	Upper bound		
*Z*1	Control	2.014	0.023	1.985	2.042	1.99	2.04
	Trios 3A	2.018	0.037	1.971	2.064	1.96	2.06
	Trios 3B	1.998	0.047	1.938	2.057	1.96	2.08
	Dental Wings	1.990	0.063	1.911	2.068	1.88	2.04

*Z*2	Control	3.986	0.011	3.972	4.001	3.97	4.00
	Trios 3A	3.988	0.072	3.898	4.077	3.88	4.05
	Trios 3B	3.952	0.073	3.861	4.042	3.90	4.08
	Dental Wings	3.97	0.054	3.902	4.037	3.89	4.02

*Z*3	Control	6.15	0.137	5.979	6.32	5.97	6.32
	Trios 3A	5.906	0.209	5.645	6.166	5.71	6.19
	Trios 3B	5.978	0.011	5.964	5.991	5.96	5.99
	Dental Wings	6.142	0.155	5.949	6.335	5.92	6.31

*Z*4	Control	8.046	0.04	7.995	8.096	8.01	8.11
	Trios 3A	7.968	0.085	7.861	8.074	7.86	8.04
	Trios 3B	7.978	0.136	7.808	8.147	7.84	8.14
	Dental Wings	7.996	0.158	7.799	8.192	7.72	8.11

SD = standard deviation; CI = confidence interval for mean; min = minimum; max = maximum.

**Table 5:** Multiple comparison of the various measurements in the *X*-axis of control with other scan groups (Trios 3A, Trios 3B, and Dental Wings).

Measurements		Comparison groups	Mean difference	*P* value
*X*1	Control	Trios 3A	0.062	0.190
		Trios HD	0.052	0.322
		Dental Wings	0.062	0.190

*X*2	Control	Trios 3A	0.074	0.082
		Trios HD	−0.03	0.727
		Dental Wings	0.072	0.093

*X*3	Control	Trios 3A	0.118	0.299
		Trios HD	−0.062	0.777
		Dental Wings	0.046	0.893

*X*4	Control	Trios 3A	0.194	0.309
		Trios HD	0.212	0.240
		Dental Wings	−0.202	0.277

*X*5	Control	Trios 3A	0.17	0.719
		Trios HD	−0.18	0.683
		Dental Wings	−0.802	0.001^*∗*^

*X*6	Control	Trios 3A	0.548	0.143
		Trios HD	0.248	0.737
		Dental Wings	−1.336	<0.001^*∗*^

^*∗*^Significant difference at *P* value <0.05.

**Table 6:** Multiple comparison of the various measurements in the *Y*-axis and the *XY*-axis of control with other scan groups (Trios 3A, Trios 3B, and Dental Wings).

Measurements	Comparison groups		Mean difference	Sig.
*Y*1	Control	Trios 3A	0.076	0.092
		Trios 3B	0.078	0.081
		Dental Wings	0.104	0.015^*∗*^

*Y*2	Control	Trios 3A	0.07	0.711
		Trios 3B	0.066	0.746
		Dental Wings	0.076	0.657

*Y*3	Control	Trios 3A	0.224	0.637
		Trios 3B	0.124	0.910
		Dental Wings	0.298	0.407

*Y*4	Control	Trios 3A	0.188	0.929
		Trios 3B	−0.024	1.000
		Dental Wings	0.408	0.567

AR	Control	Trios 3A	0.254	0.692
		Trios 3B	0.002	1.000
		Dental Wings	−0.918	0.005^*∗*^

AL	Control	Trios 3A	−0.336	0.402
		Trios 3B	−0.314	0.459
		Dental Wings	−0.848	0.004^*∗*^

^*∗*^Significant difference at *P* value <0.05.

**Table 7:** Multiple comparisons of the various measurements in the *Z*-axis of control with other scan groups (Trios 3A, Trios 3B, and Dental Wings).

Measurements	Comparison groups		Mean difference	Sig.
*Z*1	Control	Trios 3A	−0.004	0.999
		Trios 3B	0.016	0.957
		Dental Wings	0.024	0.872

*Z*2	Control	Trios 3A	−0.002	1.000
		Trios 3B	0.034	0.836
		Dental Wings	0.016	0.979

*Z*3	Control	Trios 3A	0.244	0.119
		Trios 3B	0.172	0.366
		Dental Wings	0.008	1.000

*Z*4	Control	Trios 3A	0.078	0.766
		Trios 3B	0.068	0.831
		Dental Wings	0.050	0.923

^*∗*^Significant difference at *P* value <0.05.

## Data Availability

The data used to support the findings of this study are available from the corresponding author upon reasonable request.
